# Cardiovascular emergencies in primary care: an observational retrospective study of a large-scale telecardiology service

**DOI:** 10.1590/1516-3180.2017.0090110617

**Published:** 2017-11-06

**Authors:** Milena Soriano Marcolino, Thales Matheus Mendonça Santos, Fernanda Cotrim Stefanelli, João Antonio de Queiroz Oliveira, Maíra Viana Rego Souza e Silva, Diomildo Ferreira Andrade, Grace Kelly Matos e Silva, Antonio Luiz Ribeiro

**Affiliations:** I MD, PhD. Adjunct Professor, Department of Internal Medicine, Medical School, and Telehealth Center, University Hospital, Universidade Federal de Minas Gerais (UFMG), Belo Horizonte (MG), Brazil.; II MD. Radiology Resident, Telehealth Center, University Hospital, Universidade Federal de Minas Gerais (UFMG), Belo Horizonte (MG), Brazil.; III MSc. Pharmacist and Doctoral Student, Medical School and Telehealth Center, Universidade Federal de Minas Gerais (UFMG), Belo Horizonte (MG), Brazil.; IV Medical Student, Telehealth Center, Universidade Federal de Minas Gerais (UFMG), Belo Horizonte (MG), Brazil.; V Pharmacist, Telehealth Center, University Hospital, Universidade Federal de Minas Gerais (UFMG), Belo Horizonte (MG), Brazil.; VI MD, PhD. Full Professor, Department of Internal Medicine, Medical School, and Telehealth Center, Universidade Federal de Minas Gerais (UFMG), Belo Horizonte (MG), Brazil.

**Keywords:** Emergencies, Primary health care, Cardiovascular diseases, Telemedicine, Electrocardiography

## Abstract

**BACKGROUND:**

Electrocardiograms (ECGs) are an essential examination for identification and management of cardiovascular emergencies. The aim of this study was to report on the frequency and recognition of cardiovascular emergencies in primary care units.

**DESIGN AND SETTING:**

Observational retrospective study assessing consecutive patients whose digital ECGs were sent for analysis to the team of the Telehealth Network of Minas Gerais.

**METHODS:**

Data from patients diagnosed with cardiological emergencies in the primary care setting of 750 municipalities in Minas Gerais, Brazil, between March and September 2015, were collected via telephone contact with the healthcare practitioner who performed the ECG. After collection, the data were subjected to statistical analysis.

**RESULTS:**

Over the study period, 304 patients with cardiovascular emergencies were diagnosed within primary care. Only 73.4% of these were recognized by the local physicians. Overall, the most frequent ECG abnormalities were acute ischemic patterns (44.7%) and the frequency of such patterns was higher among the ECGs assigned as emergency priority (P = 0.03). It was possible to obtain complete information on 231 patients (75.9%). Among these, the mean age was 65 ± 14.4 years, 57.1% were men and the most prevalent comorbidity was hypertension (68.4%). In total, 77.9% were referred to a unit caring for cases of higher complexity and 11.7% of the patients died.

**CONCLUSION:**

In this study, cardiovascular emergencies were misdiagnosed in primary care settings, acute myocardial ischemia was the most frequent emergency and the mortality rate was high.

## INTRODUCTION

Cardiovascular diseases are the main cause of mortality in the world, representing 32% of deaths worldwide in 2015.^1^ Following the global trend, cardiovascular diseases are also the leading cause of mortality in Brazil, representing 27% of all deaths in 2014.^2^ Because of the high socioeconomic impact and high morbidity and mortality of chronic conditions, diagnosis, management and follow-up of these patients is a priority for the Brazilian national healthcare system (Sistema Único de Saúde, SUS).^3^^,^^4^

The Brazilian public healthcare system is composed of a hierarchical structure made up of three levels dealing with cases of increasing complexity: primary, secondary and tertiary care. The aim of the system is to provide integrated universal (free access) care to meet the healthcare needs of the Brazilian population.^5^ Thus, primary care is the basis of the healthcare system and acts as a gatekeeper.^6^ This setting is responsible for assessment of diseases and risks in a specific region and for developing long-term action plans.^7^ In emergency cases, two types of facility have been set up to receive patients: emergency care units, which deal with cases of intermediate complexity and are usually available in medium-size cities; and local hospitals, which are settings for dealing with cases of high complexity and are usually available in larger cities.^8^

Regarding transportation of patients to proper facilities, Brazil used the French mobile emergency medical service as a template for developing its own mobile emergency medical service (Serviço de Atendimento Móvel de Urgência, SAMU). Currently, this model comprises a nationally standardized medical service that provides transportation and pre-hospital care for emergencies at any time on any given day.^8^

However, patients with emergencies sometimes seek assistance within the primary care setting for a variety of reasons. Firstly, the emergency services still suffer from highly restricted capacity. There is a lack of ambulances and healthcare professionals, especially in small and remote municipalities. Secondly, availability and accessibility at the community level make primary care the first contact point, sometimes even for emergency care. Lastly, some patients purposefully avoid emergency departments.

There is a lack of studies on emergencies within the primary care setting in developing countries.

## OBJECTIVE

The objective of this study was to report on the frequency and recognition of cardiovascular emergencies in primary care units, using the database of a public telehealth service.

## METHODS

### Study design and subjects

This was an observational retrospective study in which consecutive patients whose digital electrocardiograms (ECGs) were sent for analysis to the team of the Telehealth Network of Minas Gerais (TNMG) were assessed. This study used data from patients diagnosed with cardiological emergencies between March 2015 and September 2015.

The TNMG is a Brazilian public telehealth service that was formed through a partnership among seven public universities in 2005.^9^ Given the impact of cardiovascular diseases, the unequal concentration of medical specialists that prevails in large urban centers and the lack of electrocardiogram machines in remote municipalities, the service initially focused on tele-electrocardiography.^10^^,^^11^ For patients assisted at primary care units in small and remote municipalities, their digital ECGs are recorded and transmitted through the internet to the TNMG center for remote analysis by a specialized team. This service currently provides support for primary healthcare settings in 780 out of the 853 municipalities in the state of Minas Gerais, Brazil. This project has proved to be cost-effective and has prevented unnecessary referral to services in other municipalities.^12^

To record ECGs, the local healthcare practitioner uses special software that allows the examination to be linked to the patient’s clinical data (height, weight, risk factors, medications in use, signs and symptoms) in order to increase the accuracy of the interpretation. After the digital ECG has been recorded, it is uploaded in the TNMG website (http://www.telessaude.hc.ufmg.br). Subsequently, the examination is received by the TNMG analysis center, where it is distributed to on-duty cardiologists who are responsible for analyzing the ECGs in accordance with standardized criteria. All examinations sent for analysis to TNMG on weekdays before 8:00 pm are evaluated on the same day, while others are dealt with on the next business day.

**Figure 1.**
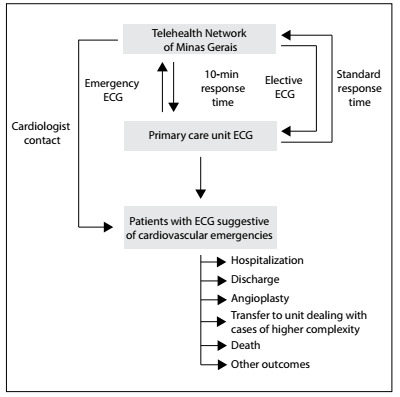
Flowchart of patient and electrocardiogram (ECG) management and outcomes.

The primary care practitioner who records the ECG can select the priority level: emergency or elective. Emergency examinations are analyzed within 10 minutes, while elective ones are assessed within 4 hours. An on-duty nursing technician is responsible for the operational management of clinical services. After a TNMG cardiologist has diagnosed a cardiological emergency situation based on the clinical data and the ECG, the nursing technician contacts the primary care unit to make a teleconsultation via internet or telephone call to guide the immediate management of the case (Figure 1). If needed, the cardiologist can request additional traces to analyze dynamic electrocardiographic changes, from the attending primary care physician.

For the purposes of this study, ECG abnormalities suggestive of acute myocardial ischemia, Mobitz II atrioventricular block, complete heart block, ventricular tachycardia, atrial fibrillation, atrial flutter with fast ventricular response, supraventricular tachycardia (atrioventricular nodal reentrant tachycardia or atrioventricular reciprocating tachycardia) and other electrocardiographic signs consistent with the clinical information were considered to be cardiological emergencies. The acute ischemic patterns included:

1. Symmetrical T-waves with increased amplitude, or symmetrical and pointed T-wave inversion;
2. ST segment elevation of at least 1 mm at the J-point, with superior convexity in this segment, in two contiguous leads that explored the region involved, for all leads other than leads V1-V3; and for leads V1-V3, ST-segment elevation in females ≥ 1.5 mm, in men over 40 years ≥ 2.0 mm and in men younger than 40 years ≥ 2.5 mm; and
3. Depression of the J-point and ST segment, such that it is horizontal or descending ≥ 0.5 mm in two contiguous leads, measured 60 ms after the J-point.^13^

### Data collection

Five trained researchers used a standardized questionnaire to collect patient data through telephone contact with the healthcare professional who performed the ECG in the primary care unit. The survey covered the following topics:

- Medications and comorbidities, when this information had not been supplied;
- Management after diagnosis (pharmacotherapy used in the primary care unit, referral to a unit dealing with cases of higher complexity, hospitalization, pacemaker implantation, thrombolysis and/or angioplasty);
- Outcome (or death).

In some of the cases, it was possible to contact the patient directly, and the same questionnaire was applied to them. Additionally, demographic and socioeconomic aspects of the patients’ municipality of origin were also assessed.^14^ The human development index (HDI) was used to provide socioeconomic information on the municipalities. This index comprises three indicators: life expectancy at birth, years of schooling and gross national income (GNI) per capita.^15^

### Statistical analysis

Categorical variables were presented as absolute and relative proportions and quantitative variables as central trend measurements and variability. The associations between variables were assessed using the chi-square test or Fisher’s exact test, when indicated, for categorical variables; Student’s t test for quantitative variables with normal distribution; and the Mann-Whitney test for quantitative variables without normal distribution. The normality of the quantitative variables was evaluated by means of the Kolmogorov-Smirnov test. The significance level for all tests was taken to be 0.05. Data management and statistical analyses were performed using the IBM SPSS software for Windows, version 21.0 (IBM Corp, Armonk, NY, USA). This study was approved by the Research Ethics Committee of the Federal University of Minas Gerais.

## RESULTS

During the study period, ECGs on 260,879 patients were recorded and then analyzed by the cardiologists of the TNMG, and 304 of them (0.1%) were diagnosed as emergencies. Considering this sample, 71% had been classified as an emergency in the primary care setting and 29% as an elective examination (which the primary care physician had not recognized as a cardiology emergency).

The total frequency of electrocardiographic abnormalities and the frequency stratified according to priority are presented in Table 1. Acute ischemic patterns were the most frequent finding, comprising 44.7% of all the cases, followed by atrial fibrillation or flutter with high ventricular response (20.6%). The frequency of acute ischemic patterns was statistically higher in the ECGs for which the priority was assigned as emergency than in those assigned as elective (48.4% versus 34.5%, P = 0.03), while the frequency of second-degree atrioventricular block was statistically higher in the ECGs with elective priority (12.3%) than in those with emergency priority (4.4%) (P = 0.03). More than one main ECG finding was observed in 24 of these 304 patients (7.9%). A combination of acute ischemic patterns and atrial fibrillation or flutter with high ventricular response was observed in 13 patients (4.3%). Other less common combinations included: ischemic patterns plus complete atrioventricular block (n = 2); ventricular tachycardia (n = 2); Mobitz II atrioventricular block (n = 2) or supraventricular tachycardia (n = 2); and atrial fibrillation plus complete atrioventricular block (n = 1) or supraventricular tachycardia (n = 2). Regarding secondary abnormalities, the most common ones were left anterosuperior divisional block (15.4%) and right bundle branch block (15.4%). When ECGs with the two priorities were compared, right bundle branch block was more frequent in the ECGs with elective priority (23.4%) than in those with emergency priority (12.5%) (P = 0.03).

**Table 1.**
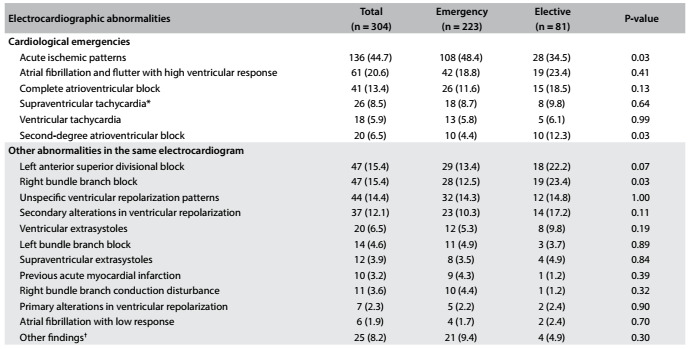
Frequency of electrocardiographic abnormalities and their stratification according to whether these were recognized as emergencies by the primary care practitioner

*Supraventricular tachycardia: atrioventricular nodal reentrant tachycardia or atrioventricular reciprocating tachycardia. ^†^Other findings included sinus arrhythmia, first-degree atrioventricular block, sinus bradycardia, atrial fibrillation, defective pacemaker, left ventricular aneurysm, severe bradycardia, marked sinus bradycardia with junctional escape beat, atrioventricular conduction disturbance, left bundle branch conduction disorders, cardiac axis deviation to the right, pathological Q waves, poor progression of the R wave, and pacemaker, with prevalence < 1.0% each.

It was possible to obtain complete information on 231 patients (75.9%) by telephone. Of these, 57.1% were men, and the mean age was 64.9 ± 14.4 years. The most common comorbidity was hypertension (68.4%) and the most frequently used drug classes were diuretics (36.3%) and angiotensin-converting enzyme inhibitors or angiotensin receptor blockers (35.5%). Only 11.7% of the patients did not use any drugs until the day of the examination (Table 2).

**Table 2.**
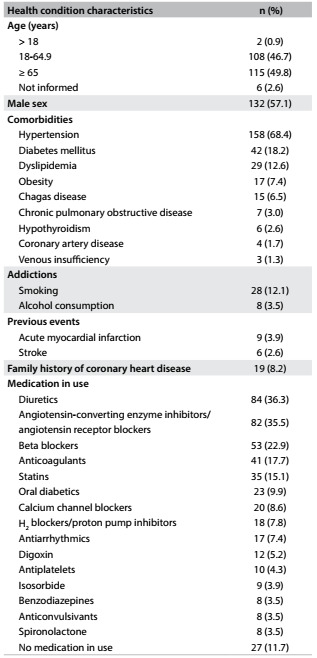
Self-declared health conditions of the patients with cardiovascular emergencies (n = 231)

With regard to management, the most frequent measures taken were referral to a unit dealing with cases of higher complexity (77.9%) and referral to a cardiologist (27.7%). Other measures included: hospitalization (27.3%), cardiac catheterization (13.9%) with or without percutaneous coronary intervention (PCI; in total, 8.2% underwent PCI), pacemaker implantation (11.7%), pharmacotherapy (6.5%), additional work-up (3.9%), referral to an intensive care unit (1.7%) and refusal of treatment (1.7%). This distribution was relatively similar when stratified according to the most frequent ECG diagnosis (Table 3). In total, 11.7% of the patients died: 10.4% of the patients with atrial fibrillation or flutter with accelerated ventricular response; 11.1% of patients with complete atrioventricular block; 14.5% of the patients with acute ischemia; and 13.3% of the patients with ventricular tachycardia. No patients with supraventricular tachycardia died.

**Table 3.**
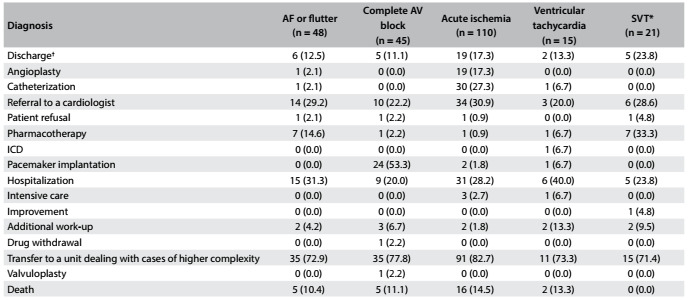
Frequency of management after diagnosis and outcomes stratified by the most prevalent electrocardiographic diagnoses observed

Values shown are n (%). AF = atrial fibrillation; AV = atrioventricular; ICD = implantable cardiac defibrillator; SVT = supraventricular tachycardia. *Supraventricular tachycardia refers to atrioventricular nodal reentrant tachycardia or atrioventricular reciprocating tachycardia. ^†^The patient was discharged immediately after the ECG diagnosis.

Among all the ECGs that the primary care practitioners classified as having elective priority, it was possible to obtain information regarding the outcome in 60 cases (74.1%). The most common outcomes were: referral to a unit dealing with cases of higher complexity (80.0%) and referral to a cardiologist (35.0%). It should be noted that it was possible to have more than one outcome for each patient: for example, a patient could firstly be referred to a unit dealing with cases of higher complexity and then be referred to a cardiologist at discharge.

Considering the social and economic data on the municipalities where the examinations were performed, the median human development index (HDI) was 0.667 (interquartile range 0.638-0.696), the median percentage of the population in poverty was 15.9% (interquartile range 7.9-27.5) and in extreme poverty, 3.9% (interquartile range 1.4-11.13) and the median population per municipality was 9,014 inhabitants (interquartile range 5,594-16,399).

## DISCUSSION

This study demonstrated that there were patients with cardiovascular emergencies who sought assistance within primary care. In 29% of these cases, the physicians did not recognize these life-threatening conditions, i.e. these were situations of misdiagnosis. The most prevalent electrocardiographic abnormalities in cardiovascular emergencies in these Brazilian primary care centers were acute ischemic patterns, followed by atrial fibrillation and flutter with high ventricular response, complete atrioventricular block, ventricular tachycardia and second-degree branch block.

Provision of emergency care is an integral part of general practice. In a review on the management of emergencies in general practice, Ramanayake et al.^16^ stated that “provision of timely, effective, proper and compassionate care requires knowledge, proper training, confidence, experience, trained supportive staff, equipment and medications”. Primary care physicians should be able to carry out at least initial pre-hospital management.^16^ However, there is a lack of good evidence on the topic of emergency preparedness.

A survey showed that only 19% of family physicians had been trained in pediatric advanced life support.^17^ To the best of our knowledge, there are no studies on training in adult advanced life support. In this light, lack of training is one of the reasons that may explain the lack of recognition of almost one third of cardiovascular emergencies. Although the patients’ clinical data was generally not provided by the primary care practitioner, we hypothesized that the severity of the cases of patients with critical presentation and poorer prognosis was more easily recognized. This could explain our observation of higher mortality rates among patients whose severe conditions were promptly recognized by the clinician (who then requested higher ECG priority).

In Brazil, periodic training in emergency care is not the reality within primary care. It is a challenge for primary care physicians to be up-to-date and competent in every emergency that they may face in the primary care setting, taking into account the wide spectrum of problems and the rarity of some of the emergencies encountered.^18^ The whole team should be trained to manage such cases and each primary care practitioner should be familiar with his/her own role in the team.^16^ The ERICO study highlighted the importance that primary care has in relation to receiving cardiovascular emergency cases and managing the pre-hospital care for these cases. In that study, 1,085 patients with acute coronary syndrome who were admitted to the university hospital of the University of São Paulo were assessed and it was demonstrated that patients who sought primary care assistance first were more likely to receive early aspirin treatment (within 3 hours) than were those who came directly to the hospital. On the other hand, 24.4% of the study participants did not receive aspirin until arriving at the hospital, although this medication is available within primary care.^19^

With regard to management of emergency conditions within primary care, some of them, such as acute asthma attacks or hypoglycemia, could be managed entirely within the primary care setting, but most of the cardiovascular emergencies needed to be transferred following initial management.^16^ In the present study, it was expected that all patients would be transferred to a unit dealing with cases of higher complexity, but this did not happen, even in cases of suspected acute coronary syndrome and third/Mobitz II second-degree atrioventricular block. Early diagnosis and management of these conditions are of utmost importance for patients’ survival.^20^

Liddy et al.^21^ reported that cardiovascular diseases were the most frequent in-office emergencies seen within primary care (32.7%). These authors suggested that it was necessary to implement guidelines regarding emergencies within primary care. In Brazil, there are national guidelines for management of emergencies within primary care, but obstacles to their implementation continue to exist.^22^ The current guidelines are not ideal, because they do not consider the regional particularities of Brazil or the social inequalities in this country.

We believe that telehealth is a low-cost and effective strategy for supporting primary care practitioners, especially those located in remote towns.^23^ Telehealth helps to improve the quality of care and reduce socioeconomic inequalities relating to access to specialized care,^24^ through providing analysis on examinations and teleconsultations (second opinions). In the present study, the physicians could have contacted the telehealth service to seek assistance in management of their patients, through online teleconsultations. Additionally, telehealth may also be helpful through providing continuing distance education courses.^25^

The results from this study have been presented to the state’s health department and have led to development of some actions by the TNMG: an e-book is being produced, to explain to primary care physicians what the meaning and management of each ECG abnormality is; an online lecture on diagnosis and management of myocardial ischemia was produced; online lectures on atrial fibrillation have also been produced and an anticoagulation management system has been developed.

This study has some limitations. It was based on the emergencies detected through electrocardiogram analyses performed by cardiologists of the TNMG. The patients were from 780 different municipalities spread out across Minas Gerais, Brazil, a state that is as large as France. Unfortunately, it was not possible to perform a before-and-after study, since the service was implemented in these municipalities some years ago and there is no unified electronic register in the Brazilian health system. All the information gathered for this study was obtained through telephone contacts with the healthcare professionals who attended these patients in the primary care setting or from the TNMG system. The system made available the patients’ ECGs tracings and clinical information that had been provided through anamnesis and which was forwarded together with the ECGs. For the same reason, it was not possible to assess whether the patients classified as emergencies were truly emergencies, or the degree of severity of these patients’ conditions. Additionally, the outcomes could not be fully assessed, since the management of cardiovascular emergencies in remote and small municipalities in Brazil includes referral to settings that deal with cases of higher complexity. Once these patients have been admitted to such centers, they are seen by different healthcare practitioners and it was therefore difficult to obtain further clinical information. The primary care practitioners reported data on comorbidities and medications, and this left room for underreporting or misreporting. It was not possible to access the whole sample of patients with cardiovascular emergencies that were diagnosed at that time, due to difficulties in contacting the primary care practitioners. Hence, patient mortality may have been underestimated.

## CONCLUSION

In conclusion, cardiovascular emergencies are misdiagnosed within primary care settings in Brazil, and acute myocardial ischemia is the most frequent emergency. There was a high mortality rate, but it was even higher among patients with acute ischemia and ventricular tachycardia.
